# Evaluation using artificial intelligence shows post pandemic differences in oral reading fluency between Brazilian public and private school students

**DOI:** 10.1038/s41598-025-15644-y

**Published:** 2025-08-17

**Authors:** Thiago Wendt Viola, Mônica Timm de Carvalho, Rita Carolina Pozzer Krumenauer Padoin, Adriana Justin Cerveira Kampff, Alexandre Vontobel Padoin

**Affiliations:** 1 Graduate Program in Medicine and Health Sciences, School of Medicine, Pontifical Catholic University of Rio Grande Do Sul (PUCRS), Avenida Ipiranga 6690—Building 12, Room 804.1, Porto Alegre, RS Brazil; 2 Graduate Program in Education, Polytechnic School, PUCRS, Porto Alegre, RS Brazil

**Keywords:** Reading fluency, Public school, Private school, Learning outcomes, Artificial intelligence, Cognitive neuroscience, Learning and memory

## Abstract

The investigation into differences in academic achievement between private and public school students has long been a focal point. The aim of this study was to compare oral reading fluency between private and public schools in the post-pandemic period. A total of 1296 participants were recruited from various Brazilian cities, spanning from the 2nd to the 5th grade of elementary school. Utilizing an artificial intelligence Universal Language Model from the Azure SST platform, the audio files were analyzed reflecting the commonly spoken language in Portuguese, generating data on words read correctly per minute (WRCM), the percentage of correct words over incorrect words (PCW), average consecutive correct words (CCW), and average silence time between sentences (SBS). Statistical analysis involved linear mixed models followed by pairwise comparisons. The private school segment outperformed the public school segment in WRCM in the 4th grade and in PCW in the 3rd grade. Additionally, private schools were already reaching 5th-grade levels in WRCM during the 4th grade, and this effect was not evident within public schools. No differences were found regarding SBS and CCW. These findings highlight that differences between public and private schools concerning reading fluency may manifest during the early stages of elementary school.

**Supplementary Information:**

The online version contains supplementary material available at 10.1038/s41598-025-15644-y.

## Introduction

The differences in academic achievement between private and public school students have long been a subject of investigation^1^. Evidence suggests that private schools often outperform public schools on key educational outcomes, including teacher attendance^2^, lower student dropout rates^3^, a higher likelihood of students entering graduate programs^4^, and overall academic performance in math and reading skills^5,6^. Some of these advantages persist even after controlling for school, family, and student characteristics^7^, highlighting the potential for improving public school performance by addressing these factors and other relevant educational outcomes.

Reading is a multifaceted skill that requires integrating diverse linguistic-cognitive abilities^8^. This literacy skill continually evolves throughout elementary school, shaping children’s developmental trajectory by facilitating the expansion of their knowledge based on vocabulary. As reading fluency develops, the link from word-level identification to reading comprehension of more complex structures becomes faster and more efficient. Thus, assessing reading accuracy and speed to monitor children’s progression is relevant not only for estimating educational achievements, but also for identifying potential disabilities and evaluating the effectiveness of reading intervention programs^9^. Most proposals consider the number of words read correctly per minute (WRCM) and the rate of correct words over errors as crucial indicators of reading fluency^10,11^. These indicators have evidenced differences between private and public schools, revealing a faster progression towards high performance in WRCM in private schools than in public schools throughout elementary and middle school grades^6^.

In addition to the factors mentioned above that could either attenuate or exacerbate disparities between public and private schools on reading fluency, another recent consideration is the impact of the COVID-19 pandemic. Although the global effects of the pandemic have lessened since 2022, its consequences on the learning and education of children remain a subject for further research^12^. During the pandemic years of 2020 and 2021, many schools turned to online/remote education to mitigate the risks of SARS-CoV-2 contamination^13^. While online strategies allowed the continuation of educational programs during the COVID-19 pandemic, prior evidence suggests that they were not as effective as regular in-person schooling for education^14,15^. As the efficiency of remote learning relies on the availability of digital resources at a large-scale community level^16^, the potential for low effectiveness in these programs could be more pronounced in low- and middle-income countries, such as Brazil. Furthermore, data from the United Nations Children’s Fund (UNICEF) shows that in November 2020, children aged 6 to 17 lacking access to education reached a milestone of 5 million in Brazil^17^. Of these, over 4 million were registered in schools but without remote learning classes throughout 2020. Potentially, these children lacking access to remote classes were those enrolled in Brazilian public schools.

Artificial Intelligence (AI) has been used more often in education in many ways, including personalized learning, intelligent tutoring systems, and automated grading and assessment. Considering the latter, recent advancements include the development of AI-assisted tools for assessing oral reading fluency, using Language Models capable of achieving high accuracy and speed in audio transcription and speech recognition^18,19^. This innovation holds promise for several advantages, including reducing subjectivity in assessments by educators, minimizing both within- and between-reading scoring discrepancies, and delivering faster results. Also, fully automated AI scoring in oral reading tests has proven to be reliable, with multiple studies reporting strong correlations for distinct features as WRCM and error coefficients, above 0.90, between human rates and AI-generated scores for distinct features such as WRCM and error rates^20–22^.

Thus, in the current study, our primary objective was to analyze differences in reading fluency between students from public and private Brazilian schools, focusing on assessments conducted in the post-pandemic era (i.e., 2022). We used a recently developed AI-assisted method to generate data on student performance from the 2nd to the 5th grade of elementary school.

## Methods

### Sample

Participants (n = 1296) were recruited from two Brazilian states (south and northeast regions), twenty-eight cities, and thirty-eight schools representing private and public segments. Eligibility was defined as regular enrollment in elementary school, specifically from the 2nd to the 5th grade, corresponding to children aged 7 to 10 years old. The recruitment process was conducted through convenience sampling. A prior study conducted in Brazil with 9th-grade students reported that the WRCM scores for private and public schools were 149.9 and 143.8, respectively, with an average standard deviation of 20.4^6^. Based on these data, a minimum sample size of 224 participants is required to detect group differences with a statistical power (beta) of 0.90 and a significance level (alpha) of 5%. This indicates that our study had sufficient statistical power to detect differences between groups.

Table 1 shows the sample’s distribution between grades and school segments. Except for the 5th grade, the private school segment had a higher sample size than the public school segment in all other grades.

**Table 1:** Sample’s distribution between grades and segments.

	Private (n = 823)	Public (n = 473)
2nd grade students	198	62
3rd grade students	300	158
4th grade students	263	139
5th grade students	62	114

### Data acquisition and procedure

This study involved the retrospective analysis of archived data. Before accessing the data, all information was fully anonymized. The utilization of the data adhered to the terms and conditions set by the Research Ethical Committee Review Board of the Pontifical Catholic University of Rio Grande do Sul, following the approved research protocol identified as number 6.582.679. Individual consent was not required, as the artificial intelligence-assisted oral reading fluency assessment has been implemented as an educational tool in Brazilian schools. Schools utilize this tool as part of their routine academic assessments to monitor student progress throughout the school year. The assessment serves as an auxiliary evaluation method adopted by schools to support literacy development. Our research team established a formal partnership with schools to analyze retrospectively collected data of the oral reading fluency assessment for research purposes. Importantly, prior to sharing any data, each school signed an official agreement consenting to participate in the study and adhered to a standardized data anonymization process. Due to the retrospective nature of the study, the need for informed consent was waived by the Pontifical Catholic University of Rio Grande do Sul Research Ethics Committee. The data was accessed for research purposes one week after the research protocol was approved (December 22nd, 2023).

Data acquisition occurred from June 2022 to October 2022 during regular school hours as part of an assignment facilitated by the teachers. Utilizing computers or tablets equipped with functional microphones, educators organized the assessment through the online platform “Oral Fluency Test” (@Elefante Letrado). Teachers needed to ensure that students were in a quiet environment, free from any noise disruptions that could affect the accuracy of the test results. After explaining how the platform works, the students were responsible for recording their own readings. Before commencing the recording, students were allowed to read the text passage silently. Following this preparatory step, students initiated the recording process by clicking/tapping the platform’s “record” button and orally reading the text. After completing the oral reading, students concluded the task by clicking/tapping the platform’s “end record” button.

The text passages were standardized according to complexity and length for the student’s grade level. Thus, 2nd-grade students had passages with an average of 152 words, 3rd-grade students with an average of 171 words, 4th-grade students with an average of 255 words, and 5th-grade students with an average of 301 words, which aligns with target word counts used in previous read fluency benchmarks^23,24^. The audio files were transcribed by speech recognition using an AI Universal Language Model from the Azure speech-to-text (SST) platform, trained with @Microsoft-owned data. This model decodes audio to capture commonly spoken language in Portuguese and has consistently demonstrated high performance in transcribing the Portuguese language^25^. To generate a word-level hypothesis that represents the student’s reading, the system aligns its output with the reference text (i.e., the assigned passage) and categorizes each word in the reference as correctly read, incorrectly read, or skipped^18^. Additionally, the system estimates a buffer length for the next sentence segment based on pauses in speech. Within each sentence segment, it transcribes words sequentially, starting from the first word and continuing until the entire segment is processed. As each new word is added, the system produces recognized events until the complete sentence segment is transcribed^19^. The Azure SST platform has been utilized in previous studies for language data acquisition and analysis, demonstrating its reliability and effectiveness in research contexts^19,26,27^.

### Measures

The AI-assisted method provided the following oral fluency reading indicators for each transcribed audio file: (1) the WRCM, which measures the accuracy and efficiency of the student’s reading by counting the number of words correctly identified and spoken in 1 min; (2) the Percentage of Correct Words (PCW), which calculates the ratio of correctly read words to incorrectly read words, providing an overall measure of reading accuracy; (3) the Average Consecutive Correct Words (CCW), which measures the average length of sequences of correctly read words. This indicator reflects the student’s ability to maintain accuracy over extended passages and offers insights into the rhythm and expression (prosody) of their oral reading. Instead of collapsing accuracy across an entire passage, CCW captures the length of error-free runs produced by a reader, estimating the concept of “fluent-run” or “longest run” metrics^28^. Longer uninterrupted strings of correct words signal more automatized decoding and fewer breakdown repairs, making CCW a more sensitive indicator of prosody; and (4) The Average Silence Time Between Sentences (SBS) captures the pause a reader leaves between finishing one sentence and starting the next. For every sentence Azure records when it starts and how long it is spoken; the silence is simply the start time of the next sentence minus the end time of the previous one (its start time plus its duration). Skilled readers typically leave shorter, more consistent pauses, reflecting efficient syntactic grouping and breath control^29^. By averaging these pauses we obtain SBS, which also helps to indicate a prosodic facet, since appropriate pauses reflect respect for sentence boundaries and an expressive rhythm that facilitates comprehension. However, it should be noted that although both CCW and SBS are indicators of prosody, they do not fully capture the construct, as prosody also involves elements such as intonation, pauses, and syntactic grouping, which these measures do not directly assess.

### Statistical analysis

The data analysis followed a two-step process. First, a Pearson correlation matrix was generated to explore relationships among the four AI-generated indicators. Next, the effects of public versus private school segments were assessed using linear mixed models, with school segment as a fixed predictor and grade level as an additional factor. Individual schools were included as a random effect to account for variability as previously suggested by Braun et al.^30^. Pairwise comparisons between grades and school segments were conducted using the Tukey method for p-value adjustments. Line plots were created to visually depict data variations across grades and school segments. For each model, both marginal R^2^ and conditional R^2^ were reported: the marginal R^2^ represents the variance explained by the fixed effects alone, while the conditional R^2^ accounts for the variance explained by both fixed and random effects. All analyses were performed using R, with statistical significance set at *p* < 0.05.

## Results

### Correlation between oral reading fluency indicators

**Fig. 1:**
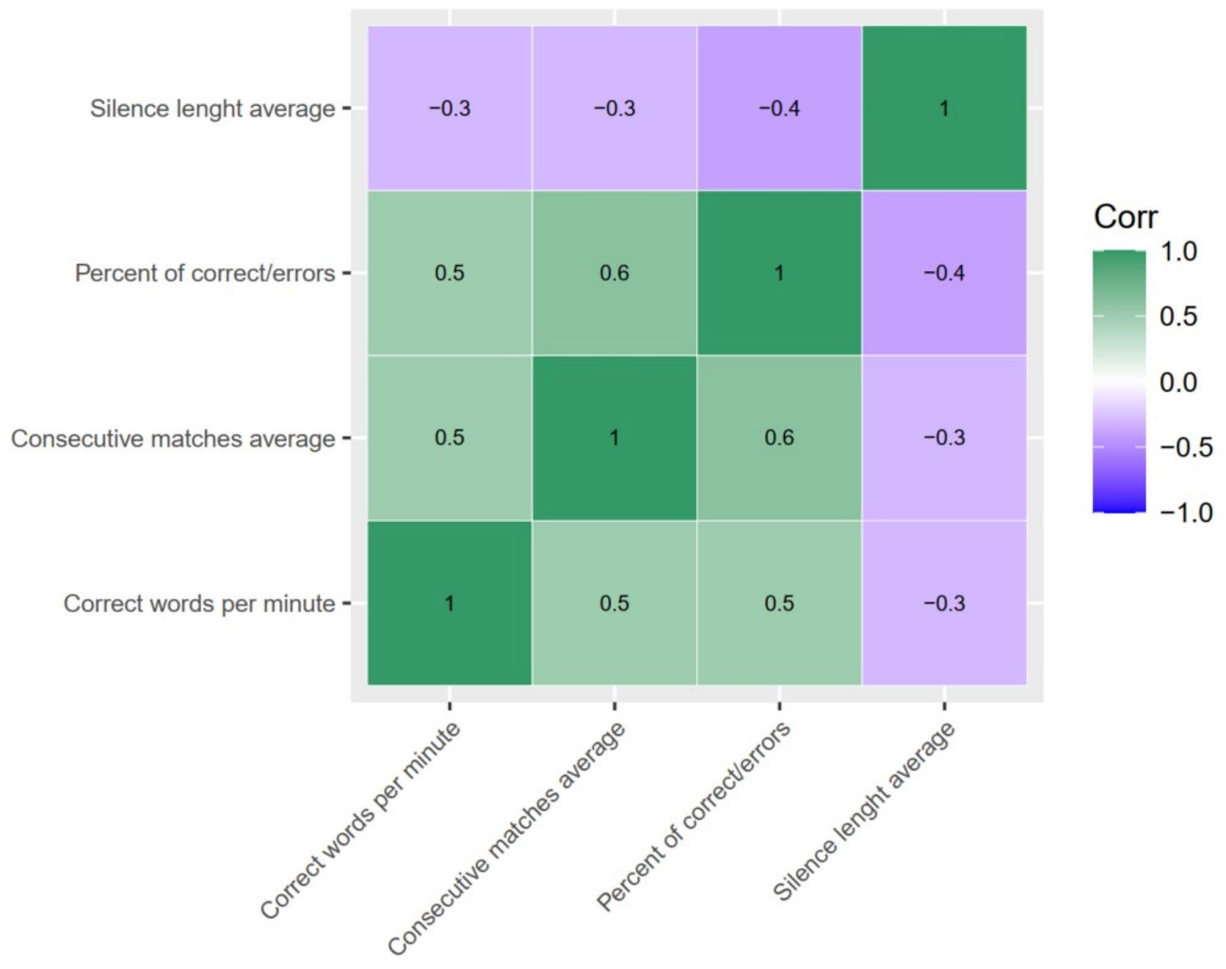
Correlation matrix between fluency reading indicators. All tested correlations were significant. Green indicates positive correlations. Purple indicates negative correlations. Color intensity indicates the strength of the correlation. Correlation coefficients are presented in the center of each square.

Correlation between indicators in all participants revealed that the WRCM positively correlated with the CCW (*p*-value < 0.001) and with the PCW (*p*-value < 0.001). The SBS metric negatively correlated with the remaining metrics (*p*-value < 0.001). The correlation coefficients are shown in Fig. 1, all indicating moderate associations with *r* values ranging from 0.2 to 0.6. These results demonstrate that the AI-assisted assessment of reading fluency revealed a specific pattern in which speed and accuracy were positively correlated, while the silence time length decreased as reading pace and accuracy improved.

### Public versus private schools

**Fig. 2:**
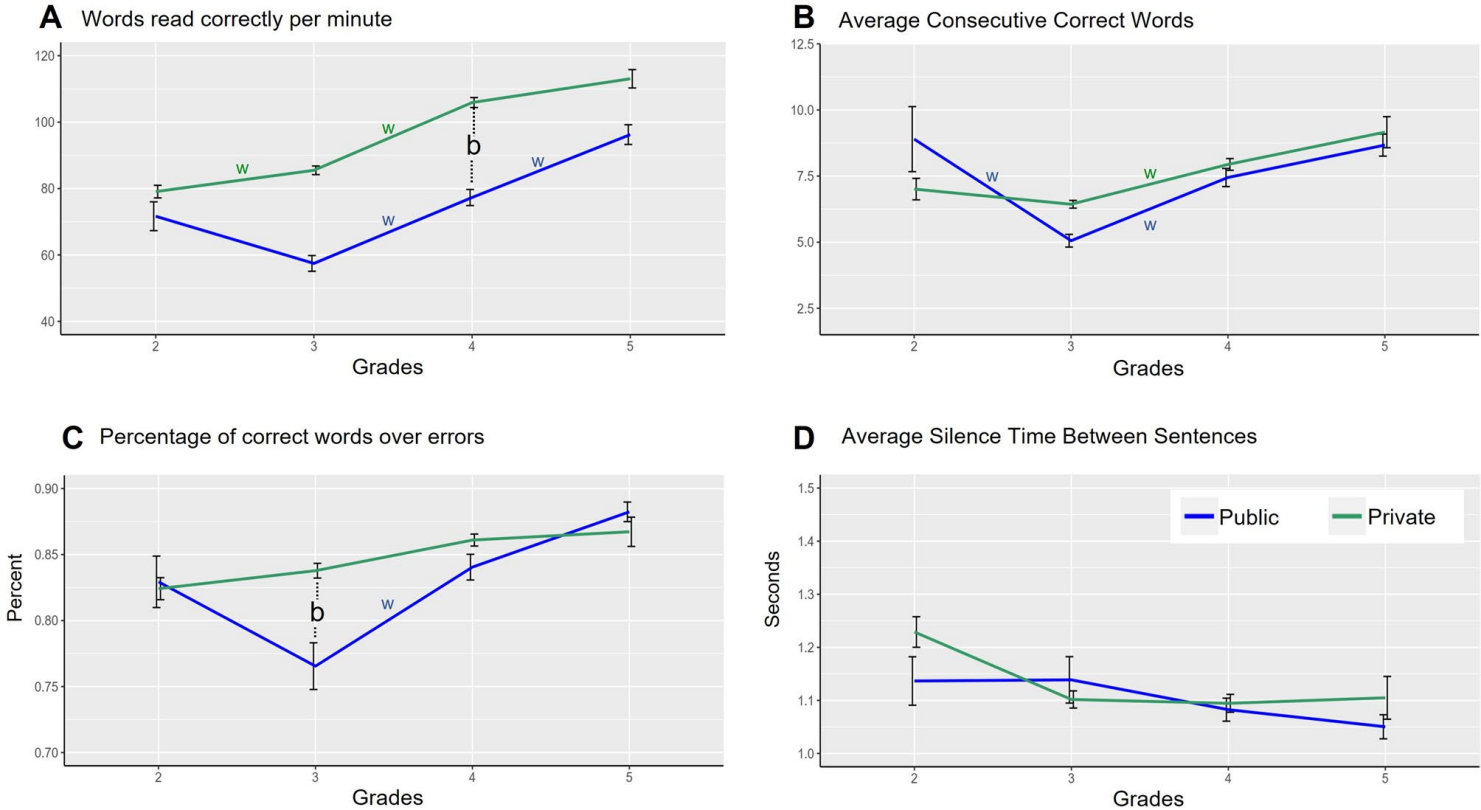
Public versus private schools performance on oral fluency reading indicators. In pairwise comparisons, w indicates a significant grade level difference within a specific school segment group. b indicates a significant school segment group difference within a specific grade level.

Next, we compared the oral reading fluency performance of elementary school students from private (n = 823) and public (n = 473) schools using linear mixed models, with school segment as a fixed predictor, individual schools as a random effect variable, and grade level as an additional factor. We observed an overall significant school segment effect for the WRCM (Marginal R^2^ = 0.20; Conditional R^2^ = 0.36; *p*-value = 0.002, Fig. 2A). To further understand this effect at each elementary school grade, pairwise comparisons were performed. The private segment outperformed the public segment in the 4th grade (*p*-value = 0.025) but not in the 2nd, 3rd, and 5th grades. Interestingly, the 3rd and 2nd grades did not differ within both segments, while the 4th grade outperformed the 3rd grade (private *p*-value < 0.001, public *p*-value < 0.001). However, no differences were detected within the private segment between the 5th and 4th grades, but these grades significantly differed within the public segment (*p*-value < 0.001). This suggests that in addition to outperforming public students in the 4th grade, private students are already reaching 5th-grade levels regarding the WRCM during the 4th grade. This effect is not evident within public schools.

A significant segment effect was observed regarding CCW (Marginal R^2^ = 0.05; Conditional R^2^ = 0.09; *p*-value = 0.026, Fig. 2B). In pairwise comparisons, however, no significant segment differences were observed in all grades. Within segment per grade comparison revealed that the 4th grade outperformed the 3rd grade in private and public segments (private *p*-value = 0.003, public *p*-value = 0.020). Still, no differences were found regarding other grades. Surprisingly, however, in the public segment, the 2nd grade outperformed the 3rd (*p*-value = 0.009), and this effect was not observed within private schools. This suggests a decrease in the performance of 3rd-grade students compared to 2nd-grade ones on CCW, as evidenced only in the public schools.

A significant segment effect was observed regarding the PCW (Marginal R^2^ = 0.07; Conditional R^2^ = 0.19; *p*-value = 0.005, Fig. 2C). In pairwise comparisons, the private segment outperformed the public segment in the 3rd grade (*p*-value = 0.028) but not in the 2nd, 4th, and 5th grades. No significant differences between grades in the private segment were revealed within the segment per grade comparison. In contrast, the 4th grade outperformed the 3rd grade (*p*-value < 0.001) in the public segment, while other grade comparisons revealed no significance. This suggests that in addition to outperforming public students in the 3rd grade, private students are already reaching considerable levels of performance regarding PCW during the initial elementary school grades. This effect is not evident within public schools since public school students kept increasing performance in the 4th grade compared to the 3rd.

No significant school segment (Marginal R^2^ = 0.02; Conditional R^2^ = 0.16; *p*-value = 0.507, Fig. 2D) was observed regarding the SBS, suggesting no evidence of public versus private school differences in this indicator. Descriptive statistics by grade level and group (including minimum, maximum, mean, standard deviation, skewness, and kurtosis) for each variable are available in supplementary materials.

## Discussion

Our study investigated whether public versus private segment could influence oral reading fluency indicators during elementary school. The assessment was conducted approximately two years after the start of the COVID-19 outbreak. The data generation process for these indicators was assisted by AI, including the following outcomes: WRCM as a speed measure, PCW as an accuracy measure, and the SBS as another speed latency measure. These fluency reading indicators have been used more frequently in previous investigations^6,31^, in particular WRCM and PCW. In addition, we also generated data on the average CCW, which we assumed as an indicator of prosody since it provides insights into the rhythm and expression of correct oral reading. For instance, prosody often involves grouping words into meaningful phrases and pausing appropriately between these phrases. Monitoring the average number of CCW read can help identify points at which students pause or encounter difficulties in maintaining the flow of speech, which may reflect challenges in navigating phrase boundaries related to prosody. We analyzed the performance in these four indicators across different grade levels, specifically from the 2nd to the 5th level of elementary school, observing that the private school segment outperformed the public school segment in WRCM in the 4th grade and in the PCW in the 3rd grade. No differences were found between school segments regarding the SBS and the CCW.

A previous study conducted in Brazil with a broader age range sample (6th to the 9th year of elementary school) demonstrated significant differences between public and private schools, particularly in the 7th grade, where private schools outperformed public schools in terms of WRCM^6^. Another study, with a more similar age range of Brazilian participants (10 to 11 years old), also found that private school students outperformed their public school counterparts in WRCM^32^. However, our findings of such differences occurring in earlier grades, such as the 4th grade, have not been consistently observed, as most studies have focused on older children. We also observed that private students are already reaching 5th-grade levels regarding the WRCM during the 4th grade, and this maturation effect is not evident within public schools. Furthermore, our findings indicated that children from private schools performed better in the PCW compared with public school students even earlier, such as during the 3rd grade. Similar early emerging gaps have been documented in other transparent orthographies. In Italy, a large survey reported an 86-point gap between high- and low-socioeconomic background children in the 4th grade on an international reading scale^33^. Comparable inequalities are already visible in Spanish, as Cubilla-Bonnetier et al. examined 2nd–3rd graders in Panama and found that the private-school group read almost three times faster and made significantly fewer errors than their public-school peers^34^.

This suggests that differences between public and private schools regarding reading fluency may emerge during the early stages of elementary school. Notably, the transition from the 3rd to the 5th grade is recognized as a crucial period marked by a shift towards the utilization of the lexical route, as opposed to the phonological route prominent in earlier years^31^. The lexical route involves the direct recognition of familiar words as whole units, relying on the reader’s stored mental lexicon, which contains a mental representation of words. Although we did not find differences in 2nd-grade performances between public and private students, potentially related to the phonological route, we cannot rule out the possibility that certain everyday practices and activities in schools might influence reading fluency performance in subsequent years. Evidence suggests that the quality and frequency of reading practices experienced in the first two years of elementary school can significantly impact a student’s level of reading fluency at the end of the 3rd year^35^. Thus, these early experiences can be decisive factors in the years that follow in the educational process.

As mentioned earlier, evidence suggests that private schools outperform public schools in terms of teacher attendance^2^, lower student dropout rates^3^, and potentially in the frequency and quality of reading activities during the early school years. These factors could be associated with the disparities in the performance of oral reading fluency. However, another factor that could be associated with our findings is the impact of the COVID-19 pandemic. Families with higher socioeconomic status may have had better resources to adapt to remote learning, including access to technology, a conducive home environment, and parental support. In contrast, students from lower-income families may have faced additional hurdles, such as limited access to online resources, a lack of quiet study spaces, and potential challenges in receiving adequate parental guidance due to various socio-economic stressors. In situations where resources are scarce and parental support is limited, learning from home may exacerbate educational disparities, further widening the gap in children’s progress. In schools, children benefit from interactions with peers and supportive adults, opportunities that may be lacking for young children whose parents face pre-existing economic hardships^36^. As the effects of the pandemic have lessened and its impact becomes clearer, it will be important to track its effects on education^37^. Understanding and addressing these family disparities during the post-pandemic era is crucial for developing effective strategies to minimize the impact of the pandemic on educational equity and outcomes.

Even though statistically significant differences were not found in all cases across grade levels, the mean scores consistently show a trend favoring private school students in most variables, except for SBS, where public school students tend to make shorter pauses. We cannot rule out the possibility that these shorter pauses in public school students reflect greater automatization of the reading process. However, the consistent overall underperformance of these students compared to their private school peers also suggests the possibility of omitting key prosodic markers.

Another relevant finding, beyond the group comparisons, is the observed correlation between WRCM, CCW, and PCW. These indicators of oral reading speed and accuracy are expected to improve together as reading skills develop. Greater fluency is typically associated with shorter pause durations between sentences, which we also observed through the negative correlations with SBS, without implying a loss of prosody. In this context, studies such as Miller and Schwanenflugel^38^ have shown that as students become more fluent readers, they reduce the processing time needed between sentences. On the contrary, less skilled readers tend to pause more frequently and for longer durations, disrupting the natural flow of reading. Additional studies in more transparent languages have also found that word reading fluency and syntactic knowledge are significantly related to the development of prosody in fifth-grade students, contributing to improvements in expression, phrasing, and the reduction of inappropriate pauses^39^.

It is worth observing that the performance of public school students slightly declined from 2nd to 3rd grade, particularly in the CCW measure. A potential explanation for this may be a cohort-specific effect related to exposure to COVID-19 school shutdowns. In Brazil, children assessed in grade 3 during 2022 had begun grade 1 in 2020, precisely when public schools were closed for the longest period and remote learning was incipient. In contrast, the 2022 2nd grade cohort started school in 2021, after many systems had resumed hybrid or in-person instruction. A study conducted in Brazil suggests that early-primary cohorts affected by the 2020 school closures experienced the greatest unfinished learning in reading, with this effect potentially being more pronounced in the public system than in the private one^40^.

We utilized AI to assist in generating reading fluency data, demonstrating its potential for large-scale application by increasing efficiency, sample size, and statistical power. AI-assisted tools offer significant advantages for assessing oral reading fluency, particularly in time savings, scalability, and the ability to conduct test–retest screenings more frequently. Traditional reading assessments rely on manual scoring and data entry, often requiring hours to complete, while AI tools can evaluate an entire class in less than 20 min, reducing both time and subjectivity in the assessment process. Crucially, these efficiency gains do not compromise accuracy, as validation studies show correlations of 0.96 between fully automated scores and expert human ratings in both U.S. and Ghanaian elementary samples^20,22^. This level of agreement exceeds the typical inter-rater reliability reported for human scorers, confirming the robustness of AI-based scoring. This scalability allows entire grades to be assessed multiple times per year, enabling more precise monitoring of children’s reading progress and difficulties. These advantages are supported by a previous large-scale, multi-site study that processed over 100 h of children’s speech using AI-assisted automated extraction of fluency features, reporting this approach as scalable, automated, and cost-effective^41^. Future longitudinal studies using AI-assisted tools for reading fluency assessment may reveal additional sources of data variability that could be integrated into educational intervention programs.

An important limitation of our study is its cross-sectional design, with the absence of data from the pre-pandemic era. Although we speculate that the pandemic may have exacerbated the public–private educational gap, previous studies in Brazil have already documented that private school students perform better than public school students in aspects of reading fluency, and these studies^6,32^ were published before the COVID-19 outbreak. To be noted, however, is evidence showing that male students experienced significantly more reading fluency loss during a 24-week COVID-19 school closure^42^. Another important limitation is the absence of individual socioeconomic covariates and sex specification. Although previous research demonstrates that school-level factors can influence literacy achievement independently of family socioeconomic status^43^, and that teacher characteristics alone can explain nearly 10% of the variability in students’ academic achievement^44^, we cannot fully disentangle home and school contributions in the present data. Future studies should adopt a multilevel design that pairs the AI-based fluency task with detailed family-background data. Another limitation refers to differences in sample sizes between public and private segment, since we have a higher sample size in the private school segment. However, data collection took place between August and November 2022, when the COVID-19 pandemic was still disrupting Brazil’s public-school system far more than the private one. Municipal and state decrees often allowed private schools to resume in-person classes months before public schools^45^. Additionally, to run the AI-based reading task each school had to provide a quiet room with at least one networked computer. A national survey in Brazil performed in 2022 shows that while 91% of private elementary/secondary schools had computers available for student use, only 58% of public schools met this requirement^46^. Moreover, obtaining institutional consent and scheduling whole-class recordings proved markedly slower in the public sector, where many schools were still operating on hybrid timetables. This difference in sample sizes can affect the generalizability and statistical power of the study. However, in our statistical approach we included individual schools as a random-effect variable in the models, which potentially mitigated this limitation. In this sense, although we estimated the sample size, it should be noted as a limitation that the previous study used for this calculation involved 9th-grade students, while our study focused on students from 2nd to 5th grade, which may affect the precision of the estimation. Despite these limitations, the primary strengths of our investigation are multiple schools from different regions contributing data, and the innovative method of data acquisition using AI.

In conclusion, this study identified significant differences in reading fluency between public and private school students, specifically during the 3rd and 4th grades of elementary school. While our research focused on Brazilian students, similar difference in reading fluency between public and private schools have been reported in other countries^30,47–49^. The COVID-19 pandemic may have further widened these gaps, as disparities in access to technology and educational resources became more pronounced. Although the data were gathered in 2022, recent national and international monitoring confirms that reading proficiency has not rebounded to pre-pandemic levels^50,51^. Our findings therefore offer a critical baseline for evaluating the impact of subsequent recovery initiatives. To minimize these differences, intervention programs require an accurate and efficient method to assess reading fluency indicators.

## Supplementary Information

Supplementary Material 1.

## Data Availability

The datasets generated and/or analyzed during the current study are not publicly available due to ethical and privacy considerations. The data were originally collected as part of routine educational assessments in schools and subsequently shared with our research team under strict confidentiality agreements. However, the dataset may be available from the corresponding author upon reasonable request, subject to ethical approval and adherence to data privacy regulations. Inquiries should be directed to: [thiago.wendt@pucrs.br].
